# Comparison of the effect of bleaching with 15% carbamide peroxide and 35% hydrogen peroxide on flexural strength of Cention N in selfcured and dual-cured polymerization modes

**DOI:** 10.34172/joddd.2020.023

**Published:** 2020-06-17

**Authors:** Narmin Mohammadi, Soodabeh Kimyai, Yasaman Ghavami Lahij, Mahmoud Bahari, Amir Ahmad Ajami, Mahdi Abed Kahnamouei, Mehdi Daneshpooy

**Affiliations:** ^1^Dental and Periodontal Research Center, Faculty of Dentistry, Tabriz University of Medical Sciences, Tabriz, Iran; ^2^Department of Operative Dentistry, Faculty of Dentistry, Tabriz University of Medical Sciences, Tabriz, Iran

**Keywords:** Composite resins, Dental materials, Flexural strength, Tooth bleaching, Tooth bleaching agents

## Abstract

**Background.** The use of bleaching agents might result in microstructural changes in tooth structure and in restorative materials. This study compared the effects of bleaching with %15 carbamide peroxide and %35 hydrogen peroxide on the flexural strength of Cention N restorative material using the self-cured and dual-cured polymerization modes.

**Methods.** Sixty bar-shaped samples of Cention N restorative material were included in this in vitro study and assigned to three groups (n=20) randomly: control, bleaching with %15 carbamide peroxide and bleaching with %35 hydrogen peroxide. Each group was divided into two subgroups: samples polymerized in the self-cured mode and samples polymerized in the dual-cured mode. Then the flexurals trengths of the samples were determined. Two-way ANOVA was used to compare flexural strengths between the three groups in two polymerization modes, followed by post hoc Tukey test. Statisticals ignificance was defined at P<0.05.

**Results.** The difference in the mean flexural strength was significant in terms of the bleaching regimen (P<0.001), with significantly lower flexural strength in the two bleaching groups compared to the control group. However, the mean flexural strengths were not significantly different in terms of the polymerization mode applied (P=0.14).

**Conclusion.** The application of %15 carbamide peroxide and %35 hydrogen peroxide bleaching agents decreased the flexural strength of Cention N restorative material. Irrespective of the bleaching regimen, there was no significant difference in the flexural strength of Cention N between the self-curing and dual-curing polymerization modes.

## Introduction

Currently, there is an increased demand for improving the appearance and color of teeth.^1^ Since tooth bleaching is a readily available and conservative technique, many patients choose this technique to improve the esthetic appearance of their teeth.^1,2^ Hydrogen peroxide or agents that release peroxide, such as carbamide peroxide and sodium perborate, are used to whiten the discolored teeth.^3^ Different techniques are available for bleaching vital teeth and can be used in the office or at home, or with a combination of both.^4^ In contrast to home bleaching techniques, the dental office bleaching modality relies on the use of higher concentrations of these agents in shorter treatment periods.^5^

Vital tooth bleaching procedures do not result in visible macroscopic defects.^3,6^ However, the results of some studies have indicated microstructural changes in tooth structure and dental materials due to the use of tooth bleaching agents.^3,6^ It has been reported that the free radicals released from these agents during the bleaching procedure affect the polymer chains and double bonds of composite resins and the organic matrix of glass-ionomers.^5^

The flexural strength is one of the mechanical properties of restorative materials.^7^ It can, to some extent, predict the behavior of the material under stresses resulting from functional bite and parafunctional forces.^7^ Some studies have yielded conflicting results after evaluating the impact of bleaching agents on the flexural strengths of restorative materials.^7-10^ The results of previous studies with different methodologies have shown increases,^8^ decreases,^7^ and no changes^9,10^ in the flexural strengths of restorative materials after the use of hydrogen peroxide and carbamide peroxide bleaching agents. The discrepancies between the results of different studies might be attributed to differences in the bleaching agents, their pH and concentration, the bleaching regimen involved and the duration of the procedure, the environmental temperature, and the restorative material used.^5,8,11,12^

Recently, a new resin-based material, referred to as Cention N, has been introduced. This restorative material is a member of the alkasite family and contains alkaline fillers within a methacrylate resin matrix.^13^ An in vitro study showed that this material could prevent caries at restoration margins of Cl V cavities by releasing calcium and fluoride ions.^14^ Cention N is a tooth-colored material and has been proposed for direct restorations. The material is self-cured and can be placed within the cavity in bulk; however, it is possible to use the light-curing technique selectively. It has been reported that the flexural strength of this new material is comparable to that of nano-hybrid and micro-hybrid composite resins.^13^

Since the type and the chemical composition of the restorative material used determine the effects of bleaching agents on their mechanical properties,^11^ and since no study has ever evaluated the effect of bleaching agents on the flexural strength of Cention N restorative material using self-cured and dual-cured polymerization modes, the aim of the present in vitro study was to compare the effects of 15% carbamide peroxide and 35% hydrogen peroxide as bleaching agents on the flexural strength of this material using self-cured and dual-cured polymerization modes. Two null hypotheses were tested: (1) Different bleaching regimens do not affect the flexural strength of Cention N restorative material. (2) The flexural strength of Cention N is not different in self-cured and dual-cured polymerization modes.

## Methods

This in vitro study evaluated 60 bar-shaped samples (25 mm in length, 2 mm in width, and 2 mm in height)^8^ of Cention N (Ivoclar Vivadent AG, 9494 Schaan, Liechtenstein) restorative material with A2 shade. The local Ethics Committee approved the protocol of the study.

### Sample Size 

The results of a pilot study (n=5 in each group) were used to determine the sample size. Based on the results, the mean flexural strength value in group 1 (without bleaching) was 92.59±5.08 MPa, with 73.71±5.01 and 63.28±4.49 MPa in group 2 (bleaching with 15% carbamide peroxide) and group 3 (bleaching with 35% hydrogen peroxide), respectively. The sample size was determined at n=9 in each subgroup by considering α=0.05, a study power of 80%, and a difference of 10% in the mean flexural strength values between the groups. However, the sample size was increased to n=10 in each subgroup (a total of 60 samples) to increase the validity of the study.

The samples were prepared with the use of a silicone mold by following the manufacturer’s instructions. The materials were transferred into the molds with the use of a spatula and gently packed into the mold with a condenser. A transparent matrix band (Hawe Neos Dental, Bioggio, Switzerland) was placed on each mold, and then a glass slab was placed on it to produce a smooth surface. The samples were retrieved from the molds after their final setting.

In samples polymerized using the dual-cured mode, the samples were light-cured for 40 seconds with the use of DENTAMERICA light-curing unit (San Jose Ave. Industry, CA 91748, USA) at 400 mW/cm^2^ light intensity with the light-curing tip placed very close to the surface at a right angle. After retrieving the samples from the mold, they underwent light-curing procedures once again for 20 seconds from each side for complete polymerization. Subsequently, the samples were incubated in distilled water at 37ºC for 24 hours. Then the sample surfaces were subjected to a polishing procedure with medium, fine, and super-fine polishing disks, respectively (Sof-Lex, 3M ESPE Dental Products St Paul, MN 55144-1000 USA). The polished samples were cleansed in an ultrasonic unit in distilled water for 1 minute.^5^ After polishing, the samples were incubated in distilled water at 37ºC for one week for complete polymerization.^11^

The samples were randomly distributed into three groups (n=20) as follows: control (without bleaching), bleaching with 15% carbamide peroxide, and bleaching with 35% hydrogen peroxide. Each main group was subdivided into two subgroups (n=10): samples polymerized using the self-cured mode and samples polymerized using the dual-cured mode.

In the control group (group 1), the samples did not undergo a bleaching procedure and were stored in distilled water at 37ºC for two weeks.^15^

In group 2, the samples underwent a bleaching procedure with the use of 15% carbamide peroxide (Opalescence® PF Ultradent Products, South Jordan, UT, USA) 8 hours daily for two weeks.^14^

In group 3, the samples underwent a bleaching procedure with the use of 35% hydrogen peroxide (Whiteness HP, FGM Produtos Odontológicos, Joinville, SC, Brasil)^2^ in two sessions, one week apart, three times each session and for 15 minutes each time (totaling 90 minutes) by following the manufacturer’s instructions.

In groups 2 and 3, the bleaching agents were applied to sample surfaces so that all the surface of each sample was covered with an adequate amount of bleaching agent. After each bleaching process, the samples were irrigated with distilled water and incubated in distilled water at 37ºC until the next bleaching session. Fresh distilled water was used daily. Then the samples were placed on the supports and underwent flexural strength test in a universal testing machine (Hounsfield Test Equipment, Model HSK-S, Salfords, Redhill, Surrey, England) at a strain rate of 1 mm/min until the samples failed. The flexural strengths of the samples (σ) were calculated in MPa using the formula below:^11^

σ=3FL/2BH^2^

where F represents the failure load (in N), L represents the distance between the supports (in mm, 20 mm in the current study), and B and H represent the width and height of the specimen, respectively (in mm).

The data underwent statistical analyses with SPSS 16 (SPSS Inc., Chicago, IL, USA). Kolmogorov-Smirnov test was applied to evaluate the normality of data. Two-way ANOVA was used to assess the impact of the bleaching regimen and the material polymerization technique on flexural strength. Post hoc Tukey tests were applied for two-by-two comparisons of the study groups. P<0.05 was set as the level of statistical significance.

## Results

**Figure 1:**
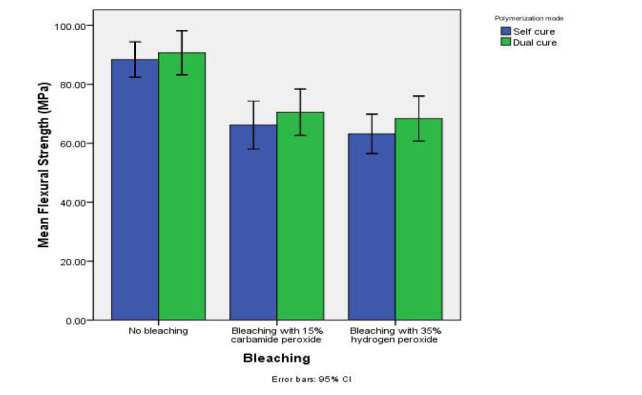
Error-bar graph of the mean flexural strength values of the study groups in terms of the bleaching regimen.

Table 1 summarizes the descriptive data on the flexural strengths of the samples and the results of comparisons made between the groups and subgroups. Figure 1 presents the error-bar graph of the mean flexural strength values in the study groups in terms of the bleaching regimen.

**Table 1:** Means and standard deviations (SD) of flexural strength values (MPa) in the study groups

**Bleaching regimen**	**Polymerization mode**	**Mean ± SD**
**Without bleaching (Control)**	Self-cured	88.38±8.37^a^
	Dual-cured	90.71±10.43^a^
**Bleaching with 15% carbamide peroxide**	Self-cured	66.16±11.37^b^
	Dual-cured	70.55±10.97^b^
**Bleaching with 35% hydrogen peroxide**	Self-cured	63.20±9.33^b^
	Dual-cured	68.38±10.68^b^

Evaluation with a post hoc Tukey test showed that mean values with the similar letter were not significantly different.

Two-way ANOVA revealed a significant difference in the mean flexural strength of the samples in terms of the bleaching regimen (F_2,54_ = 32.38, P<0.001). However, the mean flexural strength values were not significantly different in terms of the polymerization mode (F_1,54_ = 2.24, P=0.14). In addition, the interaction between the bleaching regimen and the polymerization mode was not significant (F_2,54_ = 0.10, P=0.90).

Post hoc Tukey tests showed significantly lower flexural strengths in the 15% carbamide peroxide and 35% hydrogen peroxide groups in comparison to the control (unbleached) group (P<0.001). Nonetheless, no significant difference was detected in the mean flexural strength values between the 15% carbamide peroxide and 35% hydrogen peroxide groups (P=0.70).

## Discussion

Bleaching agents can change the tooth structure, including slight surface changes, as shown under an electron microscope, and changes in microhardness and fracture toughness; in addition, they can bring about changes in the restorative materials.^3^

The current study showed that bleaching with 15% carbamide peroxide and 35% hydrogen peroxide gave rise to significant decreases in the flexural strength of Cention N restorative material with self-cured and dual-cured polymerization modes. Therefore, the first null hypothesis was refuted. In this context, a previous study showed that a 10% carbamide peroxide bleaching agent significantly decreased the flexural strengths of compomer and glass-ionomer.^11^ Another study showed that 40% hydrogen peroxide significantly decreased the flexural strength of compomer.^11^ In addition, a study reported significant decreases in the flexural strengths of composite resin, compomer, and resin-modified glass-ionomer after the application of 35% hydrogen peroxide.^7^

It has been reported that the free radicals produced during the bleaching process result in the induction of oxidative cleavage in polymer chains.^9^ Cention N is bisphenol A-glycidyl methacrylate (Bis-GMA)-free, and the organic matrix contains urethane dimethacrylate (UDMA) and low-viscosity dimethacrylates, such as trycyclodecan-dimethanol dimethacrylate (DCP) and polyethylene glycol 400 methacrylate (PEG-400 DMA).^13^ It appears that the decrease in the flexural strength after the two bleaching regimens is due to the effect of free radicals on the organic matrix of Cention N and its chemical softening.

Another reason for the decrease in the flexural strength of Cention N after the bleaching procedure might be the decrease in surface and sub-surface microhardness of the material. However, this should be evaluated in other studies. No study is available on the effect of bleaching procedures on the microhardness of Cention N restorative material. A previous study showed a decrease in microhardness of compomer and resin-modified glass-ionomer after bleaching with 10% and 15% carbamide peroxide.^16^ In addition, a decrease in the microhardness of giomer has been reported after bleaching with 15% and 45% carbamide peroxide.^15^ It was concluded in another study that 38% hydrogen peroxide, 6.5% hydrogen peroxide strips, and 5.9% hydrogen peroxide paint on bleaching products could soften the subsurface layers of universal and flowable composite resins and compomer.^17^

Cention N is an alkasite material and contains implemented alkaline fillers in the methacrylate resin matrix.^13^ The impact of the bleaching agent on the resin–filler interface in resin-based restorative materials might be another reason for the decrease in flexural strength.^11^ It has been reported that free radicals can result in the oxidation of unprotected double bonds at resin–filler interface.^8^ Besides, water sorption and partial or complete debonding has been shown due to the effect of bleaching agents on restorative materials, which might result in a decrease in hardness and integrity of these materials.^3^

In contrast to the current study, a previous study showed that 16% carbamide peroxide did not significantly change the flexural strengths of nano-filled, hybrid, and micro-hybrid composite resins.^9^ In addition, another study showed that 40% hydrogen peroxide did not decrease the flexural strength of hybrid composite resins.^10^ In the same study, an increase in the flexural strength of hybrid composite resins was reported after bleaching with 38% carbamide peroxide.^10^ The discrepancies between the results of this study and the studies above might be attributed to differences in the substrates and use of different bleaching regimens because different materials and bleaching regimens and also restorative materials might lead to contradictory results in different studies.^11^ The differences in the dimensions of the samples between this study and two previous studies^9,10^ might be another reason for the discrepancies between the results of the present study and the studies above. In the two studies above, the samples were shorter than those in the present study (12 mm), while the samples in the present study were 25 mm in length based on ISO 4049.^11^ It appears that in shorter samples the odds of flaws are lower, resulting in higher flexural strength values.

Despite the expectation that 35% hydrogen peroxide, considering its higher concentration compared to 15% carbamide peroxide, would have a more significant adverse effect on flexural strength, in the present study, no significant differences were detected between their effect on flexural strength. It has been reported that, apart from the concentration of bleaching agents, the duration of the bleaching procedure is also of importance, and the adverse effects of long-term bleaching procedures with low-concentration bleaching agents might be similar to or even more significant than short-term procedures with bleaching agents with high concentrations.^5^

Another finding of the present study was that irrespective of carrying out or not carrying out the bleaching procedure, no significant difference was detected in the flexural strength of Cention N restorative material between the self-cured and dual-cured polymerization modes. Thus, the second null hypothesis was confirmed. A study by Ilie^13^ showed that the self- or dual-cured modes had significant effects on the polymerization kinetics and conversion rates of Cention N restorative material only during the first 11 minutes of its setting. However, there were no effects on micromechanical properties, and the flexural strengths of Cention N restorative material were not significantly different between the self-cured and dual-cured modes, with their numeric value at approximately 110 MPa. It appears that the provision of proper conditions for completion of polymerization of Cention N restorative material in the self-cured mode in the present study can explain the absence of any significant differences in flexural strength between the two polymerization modes. In this study, the samples were incubated in distilled water at 37°C for one week after polishing.

Cention N is a restorative material that can release fluoride, hydroxyl, and calcium ions and can prevent the demineralization of tooth structures.^13^ Considering the advantages above, it appears that the use of this material would increase. The current study evaluated the effect of bleaching agents on the flexural strength of this material. It is suggested that in future studies the effects of bleaching agents on other physical and mechanical properties of this material be evaluated. Due to the possibility of dilution of bleaching agents in the oral cavity and the salivary buffering capacity, in vivo studies are recommended on the subject. Besides, it is advisable to study the effects of cyclic loading.

Considering the results of the present study, the clinicians should inform the patients of the possibility of changes in the flexural strength of Cention N restorative material after bleaching procedures, and the patients should be informed that after the bleaching procedure, it might be necessary to replace the restorations.

## Conclusion

The use of 15% carbamide peroxide and 35% hydrogen peroxide bleaching agents gave rise to a decrease in the flexural strength of Cention N restorative material. Irrespective of the bleaching regimen, no significant difference was noted in the flexural strength of Cention N restorative material between the self-cured and dual-cured polymerization modes.

## Authors’ contributions

The study was planned by SK, MB, NM and YGL. The literature review was performed by SK, NM, MB, AAA, MAK, and MD. SK and YGL performed the experiments and drafted the manuscript. The statistical analyses and interpretation of data were carried out by SK and NM. All the authors critically revised the manuscript for intellectual content. All the authors have read and approved the final manuscript.

## Acknowledgments

The authors would like to thank Dr. Majid Abdolrahimi (DDS), who edited the English language of this article.

## Funding

The study was sponsored by Vice Chancellor for Research at Tabriz University of Medical Sciences.

## Competing Interests

The authors declare no competing interests with regards to the authorship and/or publication of this article.

## Ethics Approval

The study protocol was approved by the Ethics Committee at Tabriz University of Medical Sciences (Ref. No. IR.TBZMED.VCR.REC.1398.096).
