# Correction to: Comparison of the effect of bleaching with 15% carbamide peroxide and 35% hydrogen peroxide on flexural strength of Cention N in self-cured and dual-cured polymerization modes

**DOI:** 10.34172/joddd.2020.045

**Published:** 2020-12-02

**Authors:** Narmin Mohammadi, Soodabeh Kimyai, Yasaman Ghavami Lahij, Mahmoud Bahari, Amir Ahmad Ajami, Mahdi Abed Kahnamouei, Mehdi Daneshpooy

**Affiliations:** ^1^Dental and Periodontal Research Center, Faculty of Dentistry, Tabriz University of Medical Sciences, Tabriz, Iran; ^2^Department of Operative Dentistry, Faculty of Dentistry, Tabriz University of Medical Sciences, Tabriz, Iran

The authors of the article entitled “Comparison of the effect of bleaching with 15% carbamide peroxide and 35% hydrogen peroxide on flexural strength of Cention N in self-cured and dual-cured polymerization modes” which appeared in *J Dent Res Dent Clin Dent Prospects* 2020; 14(2):105-109,^1^ have requested to update the Acknowledgement section of their article by adding the following sentence: “This study was supported by a grant (No. 63149) from Tabriz University of Medical Sciences.” The original version of the article has been updated to reflect these corrections.
